# Regional Variation of Bitter Taste and Aftertaste in Humans

**DOI:** 10.1093/chemse/bjz064

**Published:** 2019-09-21

**Authors:** Molly J Higgins, John E Hayes

**Affiliations:** 1 Sensory Evaluation Center, College of Agricultural Sciences, The Pennsylvania State University, University Park, PA, USA; 2 Department of Food Science, College of Agricultural Sciences, The Pennsylvania State University, University Park, PA, USA

**Keywords:** bitter taste, hops, papillae, spatial taste test, taste perception, tongue map

## Abstract

Despite widespread and persistent myths of a tongue map, all 5 prototypical taste qualities are sensed over the entire tongue. However, modern psychophysical data also suggest there may be more nuanced differences in suprathreshold intensity across oral loci, especially for bitterness. Here, we test whether bitter stimuli matched for whole-mouth intensity differ in perceived intensity across regions of the oral cavity in 2 experiments. Experiment 1 consisted of a whole-mouth sip and spit approach and Experiment 2 consisted of a spatial taste test using cotton swabs. In Experiment 1, participants (*n* = 63) rated overall intensity of 3 bitter solutions at 5 different loci (front, middle, back of tongue; roof of mouth; and lip). Temporal effects were explored using in-mouth and aftertaste ratings. In Experiment 2, participants (*n* = 48) rated the intensity of quinine and Tetralone solutions after solutions were painted on fungiform, circumvallate, and foliate papillae with a swab. After the spatial taste test, participants completed a questionnaire on self-reported beer intake. Analysis of variance results of both experiments show a significant locus by stimulus interaction, suggesting different bitterants were perceived differently across the various loci. This result was apparently driven by low-intensity ratings for Tetralone on the anterior tongue. Aftertaste ratings in Experiment 1 also revealed significant temporal effects: ratings on the anterior tongue decreased for all bitterants and ratings for quinine decreased at all loci. Reasons for these effects are not known but may suggest differential expression of bitter taste receptors or differences in bitter agonist-receptor binding affinity across tongue regions.

## Introduction

In humans, there are 25 known bitter taste receptors encoded by the *TAS2R* genes. Bitter taste receptors are activated by thousands of bitter compounds from diverse chemical classes (Belitz 1985). These compounds can activate anywhere from 1 to 15 bitter receptors, although cognate receptors have not been identified for many stimuli perceived as bitter (Meyerhof et al. 2010; Thalmann et al. 2013). Activation of bitter taste receptors during ingestion typically causes an aversive reaction. This reaction is innate and has been observed at birth in humans and primates (Steiner et al. 2001), as well as other mammals (Meyerhof 2005). In addition to the large number of bitter receptors and their structurally diverse ligands, additional complexity is added by functional polymorphisms that influence bitterness perception across individuals (e.g., Wooding et al. 2010; Allen et al. 2013; Webb et al. 2015). Indeed, the potential influence of polymorphic alleles on liking and intake of bitter foods is well documented (Hayes et al. 2013b; Running and Hayes 2016).

However, less is known about the topographical expression of bitter taste receptors. Berhens and others observed differences in expression of *hTAS2R* genes in human circumvallate papillae using in situ hybridization models (Behrens et al. 2007). Their findings suggest taste receptor cells exhibit limited capability to detect a wide range of bitter stimuli, which may lead to selective detection of bitterants. We suspect topographical differences in TAS2R expression may contribute to regional and temporal variation in the perception of bitter stimuli that has been reported previously (e.g., Collings 1974; Leach and Noble 1986; Guinard et al. 1995; Colvin et al. 2018). Our research group previously found evidence that quinine and Tetralone differ qualitatively in a sorting task (McDowell 2017); whether these differences might be due to subtle regional or temporal effects is unknown. Further, we suspect perceptual differences between bitter stimuli may influence differential liking and intake of bitter foods. Thorough exploration of this working hypothesis is predicated on documentation of clear perceptual differences between bitter stimuli.

In an early foundational study with human participants, Collings documented differential dose–response functions for quinine and urea, but only on the foliate papillae (Collings 1974). These data are consistent with the notion of intensity differences between bitter stimuli across loci, but this nuance in Collings’ data has presumably been overlooked, as her work is most often cited as a means to debunk the persistent myth of the tongue map. Although the basic premise of the tongue map is fundamentally mistaken (see Snyder et al. 2006), other psychophysical data suggest there may be some subtle regional variation in suprathreshold intensity, especially for bitterness (Collings 1974; Feeney and Hayes 2014; Colvin et al. 2018). Collectively, these data suggest that additional research is needed to explore and document differences in perceived intensity of intensity matched bitter stimuli across multiple loci on the tongue.

In humans, taste receptors are found throughout the oral cavity, including on the soft palate, pharynx, larynx, and anterior and posterior tongue. Taste buds are found in 3 types of papillae: fungiform (anterior tongue), foliate (lateral posterior tongue), and circumvallate (central posterior tongue), and vary in size and structure (Miller 1995). Taste transduction events from papillae are relayed to the brain through multiple nerves. Taste sensations from the fungiform papillae are transmitted to the brain via the chorda tympani (CT) nerve while information from circumvallate and foliate papillae occur via the glossopharyngeal (GP) nerve. Electrophysiological work in mice shows differences between CT and GP responses for various bitter stimuli (Danilova and Hellekant 2003). If the differences in neural responses observed by Danilova and Hellekant for the CT and the GP correlate to perception, this may imply differences in perceived intensity on the anterior and posterior tongue. In humans, Haase and colleagues subsequently showed that methodological differences in stimulus delivery greatly influenced the dose–response curves for certain bitter stimuli, as dorsal flow and sip and spit stimulus delivery systems activate different areas of tongue (Haase et al. 2009). Critically, in their study, quinine and caffeine did not respond equally to changes in stimulus delivery. Collectively, these results suggest 2 key points: 1) regional variation in perceived intensity may exist for a single bitter stimulus and 2) regional variation in perceived intensity may vary between bitter stimuli. Presumably, regional differences in perceived intensity across bitter stimuli may be due to differences in the expression of bitter taste receptors activated by those stimuli.

The primary goal of this work was to explore regional differences in perceived intensity of bitter stimuli using a whole-mouth sip and spit procedure (Experiment 1) and a spatial taste test (Experiment 2). Experiment 1 was primarily intended to explore regional variation between bitter stimuli in an ecologically relevant manner. We did this by asking participants to rate the intensity of stimuli at 5 regions in the oral cavity while the stimulus was in the mouth. As an exploratory aim, we also collected aftertaste ratings after the stimulus was expectorated. Experiment 2 was intended to confirm regional variation seen for 2 stimuli in Experiment 1 using a spatial taste test. As an exploratory aim, Experiment 2 also tested potential relationships between differences in perceived bitterness and intake of a widely consumed bitter beverage (i.e., beer). We hypothesized we would detect differences in perceived intensity at different loci between bitter stimuli. We also hypothesized temporal differences would be observed between the bitter stimuli, as prior work suggests some stimuli linger longer, even when intensity matched (Guinard et al. 1995; Green and Hayes 2004; Fritsch and Shellhammer 2009). Last, regarding ingestive behavior, we expected individuals perceiving hop extracts (iso-alpha-acids) as more intense to drink less pale ale style beers (as those beers contain much higher amounts of these compounds relative to beers like American-style lagers).

## Materials and methods

### Overview

Convenience samples of reportedly healthy individuals were recruited to participate in 2 laboratory studies. Potential participants who had previously indicated interest in participating in taste and smell experiments were contacted and asked to complete an online screener to determine eligibility. All relevant guidelines and regulations for research with human participants were followed, including the Declaration of Helsinki for medical research involving human subjects. Participants volunteered their time and provided informed consent (details below).

### Experiment 1: regional variation in intensity ratings collected in a whole-mouth sip and spit paradigm

#### Participants

Participants (*n* = 67; 24 men, 43 women; mean age = 30 years; range = 19–45 years) were recruited from the Pennsylvania State University campus and surrounding community (State College, PA). Participant screening criteria included no chest cold, flu, or upper respiratory conditions; nonsmoking; not pregnant or breastfeeding; no lip/tongue/cheek piercings; no known taste or smell defects; no difficulty swallowing or history of choking; not over the age of 45; not taking prescription pain medication; and no history of chronic (3+) ear infections (see Bartoshuk et al. 1996; Rawal et al. 2015). This study was exempted from full IRB review by staff in the Penn State Office of Research Protections under the wholesome foods exemption in 45 CFR 46.101(b)(6). Participants provided informed consent via a click-through yes/no question on the computer screen and were compensated with a cash payment of $5 for their time.

#### Test stimuli

Quinine monohydrochloride dihydrate (0.095 mM, SAFC Supply Solutions, St. Louis, MO), sucrose octaacetate (SOA; 44 μM, SAFC Supply Solutions), Tetralone, (0.888 mL/L, donated by Kalsec, Kalamazoo, MI), and sucrose (324 mM, purchased at retail from local supermarket) were prepared in reverse osmosis (RO) water. (Tetralone 46-122 is a commercially available hop extract that has been reduced and isomerized for stability; as supplied by the manufacturer for use by the brewing industry, it is a solution of 9.5% (w/w) tetra-hydro-isoalpha-acid in water). The stimulus concentrations used here (see Table 1) were based on intensity curves from prior dose–response studies (Reyes and Hayes 2015; McDowell 2017) using general Labeled Magnitude Scales (gLMS) (Bartoshuk et al. 2003). Here, the target intensity for all stimuli was intended to be halfway between moderate and strong (17 and 35, respectively) on a gLMS. The specific bitter stimuli were selected based on prior anecdotal observations regarding differences in the perceived intensity at different locations within the oral cavity and tongue, as well as their presence in food products to provide greater ecological relevance for the study (e.g., quinine in tonic water, iso-alpha-acids in beer). Sucrose was included as a warm-up to familiarize participants with the testing procedure and rating task. All solutions were stored refrigerated overnight and brought to room temperature (~20 °C) prior to testing. Each solution was presented in 25 mL aliquots in 30 mL plastic medicine cups with 3-digit blinding codes. Each solution was presented in duplicate (8 samples total). The sucrose samples were always presented first as a warm-up and the bitter samples were presented in counterbalanced order using a modified Williams’ design (Williams 1949).

**Table 1.** Stimuli concentrations used in Experiments 1 and 2

Stimuli	Concentration
Experiment 1	
Sucrose (practice stimuli)	324 mM
Quinine monohydrochloride	0.095 mM
Tetralone	0.888 ml/L
Sucrose octaacetate	44 µM
Experiment 2	
Quinine monohydrochloride	0.85 mM
Tetralone	7.99 ml/L

#### Study procedures

Data collection occurred in the Sensory Evaluation Center at Penn State in semi-isolated testing booths under a northern daylight illuminant (5000K LED) located directly overhead. Testing consisted of one (~30 min) testing session. All data were collected using Compusense Cloud, Academic Consortium (Guelph, Ontario, Canada).

All intensity ratings were collected using a gLMS. The gLMS is a semantically labeled line scale, with marks at 0 (“no sensation”), 1.4 (“barely detectable), 6 (“weak”), 17 (“moderate”), 35 (“strong”), and 51 (“very strong); the top anchor (100) used here was “strongest sensation of any kind” (Bartoshuk et al. 2003). Prior to rating experimental samples, participants were provided with instructions on how to use the scale and completed a warm-up exercise involving 15 remembered or imagined sensations (Hayes et al. 2013a). Both food and non-food stimuli were included in the warm-up to 1) demonstrate the scale’s applicability across multiple sensory modalities and 2) show that the scale should be used to describe all sensations within the same context. Ratings from the gLMS warm-up exercise were evaluated for each participant to determine if they used the scale correctly. A working definition of proper scale usage has been outlined previously (Nolden and Hayes 2015). Based on these criteria, 4 participants failed to use the scale correctly, and the sample intensity ratings from these participants were excluded from final analyses, resulting in a final *n* of 63. Of the 4 participants who failed to show proper gLMS usage, 3 failed to properly rank the 3 sound or light sensations in the same order (allowing for deviation of up to 5.0 units on the 100-unit scale) and 1 rated 6 of the sensations greater than 95 (on a 100-unit scale). All other participants showed evidence of appropriate scale usage in the warm-up exercise.

Following the gLMS warm-up, participants were asked to rate the overall intensity of the samples (without the use of nose clips) for 5 different oral regions: front, middle, back of tongue; roof of mouth; and lip. The lip intensity rating was included as a control for demand characteristics of the task (Orne 1962). To explore temporal differences, ratings were collected twice: once while the solution was held in the mouth, and a second rating after spitting out the solution and waiting briefly (i.e., an aftertaste rating). Intensity ratings for the sucrose stimuli were not included in the final analyses, as the sucrose solutions were intended merely as practice stimuli. Participants were instructed to place the entire sample in their mouth and to swish the samples around to cover all areas of the mouth before rating the intensity. Rating scales for all loci were presented simultaneously on a single computer screen, and the order of loci was fixed: front, middle, back of tongue; roof of mouth; and lip. The loci ratings were not randomized (i.e., always presented in the same order) as we did not want to confuse participants by presenting the scales in a different order across trials (e.g., rating the back of tongue 1st on trial 1, but 4th on trial 2, etc.), and we wanted to present the loci in the order the participant would experience the sensations (i.e., front before back). After the in-mouth intensity ratings were collected, participants were instructed to expectorate the sample and wait 15 s, without rinsing, before rating the intensity of the aftertaste on a second set of identical scales. After completing the aftertaste ratings, participants waited for a proscribed interstimulus interval (ISI), and were instructed to rinse their mouths with water at least 3 times with RO water and wait until no taste sensations persisted to proceed to the next sample. The ISI for sucrose was 60 s minimum, and this was increased to 90 s minimum for bitter stimuli; these minimums were enforced via software.

### Data analysis

All data were analyzed using SAS version 9.4 (Cary, NC). Duplicate ratings for each solution at each locus were averaged for each participant and log transformed prior to analysis (1 point was added to all intensity ratings to resolve the inability to take the log of zero). Logs of ratings were used due to the log normal distribution typically seen with this type of scaling data (Green et al. 1996). To test whether loci and/or stimuli affected bitterness intensity, repeated measures analysis of variance (ANOVA) was performed via *proc mixed* separately for in-mouth and aftertaste intensity data; stimuli and loci were considered fixed effects and participants were random. All 2-way interactions were included in the model and interpreted prior to lower order factors. If a significant locus by stimulus interaction was observed, the simple effects of both locus and stimuli were analyzed using the *slice* option within the *lsmeans* statement in SAS. If a significant simple effect was found for a specific locus or stimulus, the intensity values were compared. No corrections for multiple comparisons were made; however, Bonferroni corrections are noted in the appropriate figure captions. An α level of 0.05 was set a priori for all analyses. The analyses for aftertaste ratings were conducted using the same model as the in-mouth ratings. To test for temporal effects (i.e., differences between the in-mouth and aftertaste ratings), repeated measures mixed model ANOVAs were performed separately for each bitterant; time and locus were fixed effects, while participants were random.

## Results for Experiment 1

### In-mouth intensity ratings

**Figure 1.**
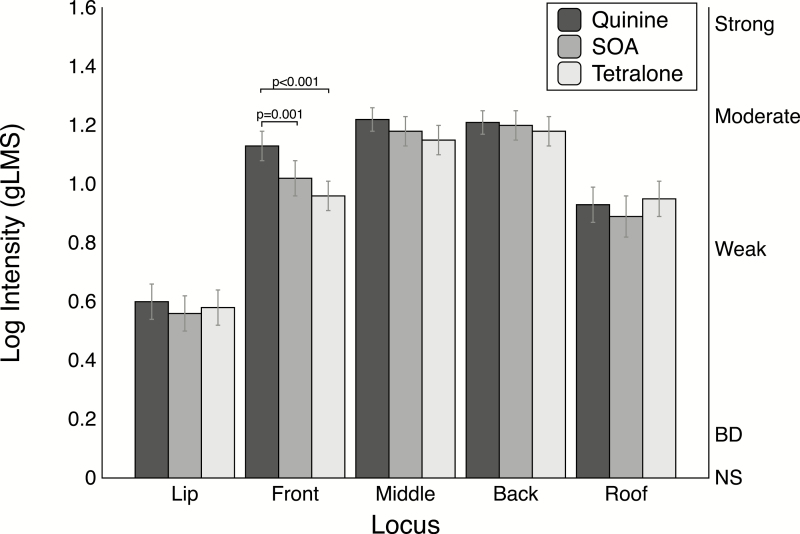
Mean log intensity ratings and standard error of the mean for in-mouth ratings at different oral loci to represent the stimulus effect from Experiment 1. The intensities between stimuli at each locus were compared using *t*-tests (see text for details). To perform a Bonferroni adjustment for multiple comparisons within a single locus, the *P*-value shown should be multiplied by 3 (e.g., *P* = 0.001 would be adjusted to *P* = 0.003).

For the in-mouth intensity ratings, repeated measures ANOVA revealed a significant interaction for locus by stimulus [*F*(8, 496) = 4.23; *P* < 0.001]. As shown in Figure 1, the solutions were generally well matched for overall intensity, and as expected, the intensity differed across locus. Generally, ratings on the back and middle of the tongue were higher than other regions. Mean ratings on the lip were lowest (below weak on a gLMS), as would be expected as the lips do not contain taste receptors (these ratings were included as a control for potential demand characteristics of the task). Decomposition of the simple effect of stimulus via the *slice* option in SAS revealed significant differences between stimuli on the front of the tongue (Figure 1). Quinine was rated significantly greater than both SOA (*t* = 3.29, *P* = 0.001) and Tetralone (*t* = 4.84, *P* < 0.001).

The *slice* option also revealed a significant effect of loci for all stimuli. Both SOA and Tetralone were rated significantly higher on the back and middle of the tongue than the other rated areas (Supplementary Figure 1). Quinine was the only stimulus that did not significantly differ on the front, middle, and back of tongue. As expected bitterness ratings for all stimuli were lower on the roof and lip.

### Aftertaste intensity ratings

**Figure 2.**
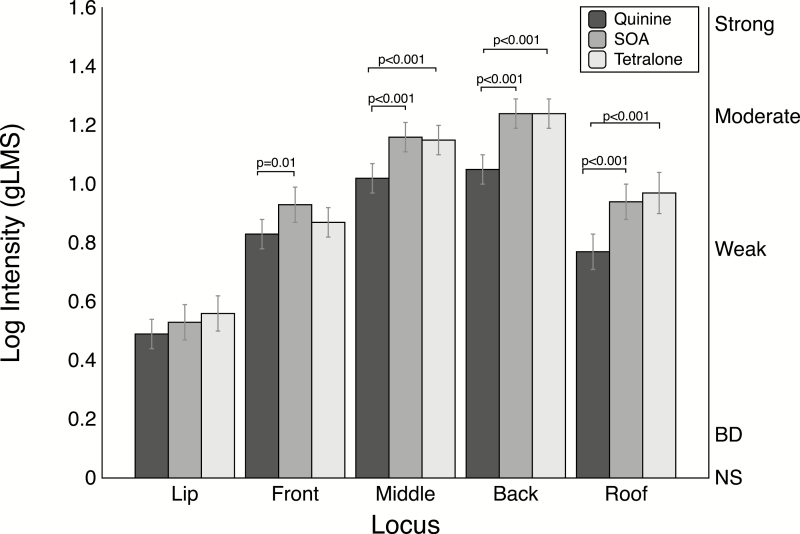
Mean log intensities and standard errors at different oral loci for aftertaste ratings made 15 s after spitting out the sample. Other details are the same as in Figure 1.

The overall rank ordering of the intensity ratings for aftertaste revealed very similar patterns to the in-mouth intensity ratings (i.e., the back of the tongue was the highest for each stimulus, the lip intensity ratings were the lowest and all below weak, etc.). For the aftertaste ratings, repeated measures ANOVA also revealed a significant locus by stimulus interaction [*F*(8, 496) = 4.37; *P* < 0.001]. The locus by stimulus interaction was further analyzed using the *slice* option as above. The simple effect of stimulus was significant for all loci except the lip, as shown in Figure 2. When the aftertaste means for each stimulus were compared at each locus, quinine was consistently less intense than both SOA and Tetralone (see Figure 2), despite having been well matched for intensity in the in-mouth tasting, suggesting it decays (cleans up) more quickly than the other 2 bitterants. The simple effect of locus was significant for all stimuli in the aftertaste ratings, as shown in Supplementary Figure 2.

**Figure 3.**
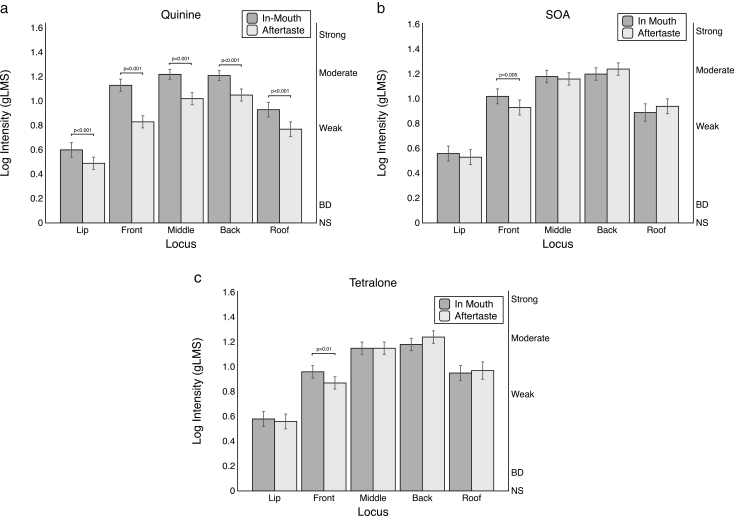
Mean in-mouth and aftertaste log intensity ratings for (a) quinine hydrochloride, (b) sucrose octaacetate (SOA), and (c) Tetralone, a hop extract, from Experiment 1. Comparisons at each locus represent *t*-tests following a significant locus by time interaction in the repeated measures ANOVA conducted separately for each stimulus (see text for details). To perform a Bonferroni adjustment for multiple comparisons across the 5 different loci, the *P*-value shown should be multiplied by 5 (e.g., *P* = 0.005 would be adjusted to *P* = 0.025).

Based on the apparent differences in decay rate across the stimuli and loci (cf. Figures 1 and 2), we tested time by locus models separately for each bitterant (Figure 3a–c). For quinine (Figure 3a), the time by locus interaction [*F*(4, 248) = 6.44; *P* < 0.001] was significant. Given the significant interaction, the means were compared at each locus. Comparison of means shows that quinine aftertaste ratings were consistently lower than the in-mouth ratings across all loci (all *t* values > 3.51). For SOA (Figure 3b), the time by locus interaction [*F*(4, 186) =4.96; *P* < 0.001] revealed only one significant difference with a lower aftertaste rating at the front of the tongue (*t* = 2.84, *P* = 0.005). For Tetralone (Figure 3c), the pattern was the same as SOA: the time by locus interaction [*F*(4, 248) = 4.60; *P* = 0.001] was significant. When the means were compared at each locus, a significant lower aftertaste intensity on the front of the tongue was observed (*t* = 6.39, *P* = 0.01). There were no other significant differences.

## Experiment 2: perceived intensity of bitter stimuli applied to discrete regions of the tongue via cotton swabs

### Materials and methods

#### Participants

Participants (*n* = 48; 24 men, 24 women; mean age = 27.4 years; range = 18–41 years) were recruited using the same criteria as in Experiment 1; 16 of these participants had also participated in the first experiment. All procedures were approved by the Pennsylvania State University Office for Research Protections (protocol #00009197). Participants provided written consent prior to participation and were compensated with a cash payment of $5 for their time.

#### Test stimuli

Given the length of time required to collect data in a spatial taste test, we wanted to reduce the total length of testing to 1) minimize burden on participants, and 2) minimize inattention and fatigue. Accordingly, only 2 bitterants from Experiment 1 were used in Experiment 2 (see Table 1). Based on results from Experiment 1, as well as their presence in the food supply, quinine HCl (0.85 mM, SAFC Supply Solutions) and Tetralone (7.99 mL/L, Kalsec) were used (i.e., SOA was dropped from testing). In an attempt to preserve the overall intensity matching of the 2 stimuli, and to account for the decrease in stimulus volume and area of stimulation, the concentrations used for the spatial taste test were 9 times the stimuli concentrations used in Experiment 1. The spatial taste was meant to confirm the results from Experiment 1 using a protocol with greater experimental control to test for effects of receptive field, acknowledging that this increase in experimental control comes at the cost of ecological validity (i.e., using a cotton swab to stimulate individual tongue regions is differs substantially from how one would typically experience these sensations during normal eating and drinking).

#### Study procedures

All testing consisted of 1 test session (~30 min) held one on one with the experimenter in a windowless clinical-style examination room in the Erickson Food Science Building at Penn State under standard white fluorescent lighting. The experimenter was seated face to face with the participant, and all data were collected on an Apple iPad Air (9.7-inch display, Apple Inc., Cupertino, CA) using Compusense Cloud, Academic Consortium (Guelph, Ontario, Canada). A modified version of the spatial taste test described previously (Bartoshuk 1989; Kveton and Bartoshuk 1994) was used to test differences in overall intensity between the stimuli across different regions of the tongue. Spatial taste tests have typically been used to assess taste damage in individuals (Bartoshuk 1989; Rawal et al. 2015), but they can also be used to explore regional differences across stimuli (e.g., Green and Hayes 2004; Feeney and Hayes 2014; Colvin et al. 2018). Each stimulus was predominately bitter; however, overall intensity was measured at each location to incorporate any other perceived sensations from these stimuli. Intensity ratings were taken (without the use of nose clips) as right–left pairs on the anterior tongue (fungiform papillae), posterior tongue (circumvallate papillae), and edge of the posterior tongue (foliate papillae) (see Supplementary Figure 3). Participants were told their assistance would be needed to reach the application locations. For the fungiform papillae, participants were asked to extend their tongue, close their lips around their tongue to provide stability, and relax their tongue (to prevent a smaller surface due to pointing the tongue). For the circumvallate papillae, participants were provided with a small gauze pad to hold and pull their tongue out and downward so the experimenter could reach circumvallate papillae on the rearmost area of the tongue. For the foliate papillae, participants used the gauze pad to hold and pull the tongue forward and use their other hand to pull back the portion of the cheek covering the side of the tongue. Participants were shown photos to demonstrate the procedure for each location (see Supplementary Figure 4). Prior to rating any test stimuli, participants practiced with application of water to either the right or left foliate papillae (i.e., the most invasive of the 3 loci) to familiarize participants with the process and tactile sensations they would experience. No response was recorded for this practice application.

To apply the test solutions, a cotton-tipped swab was immersed in a plastic medicine cup containing one of the 2 bitter solutions until no air bubbles surfaced from the cotton tip (~5 s). The saturated swab was then gently drawn across the lip of the cup to remove excess liquid, and then rolled over the surface of the tongue for approximately 3 s to deliver the tastant to the desired region. Participants were instructed to rate the intensity of the applied solution while their tongue was extended and to minimize any oral movements to prevent the stimulus from spreading to other regions. The location order was fixed within each participant to prevent stimulating the same location twice in a row, and order of locations were counterbalanced across participants to reduce any systematic bias. The stimulus order (Tetralone or quinine first) applied to each location was also counterbalanced. For each location, the right side was always stimulated first followed by a 30-s break and the application to the left side using the same stimuli. During the 30-s interstimulus break following each intensity rating, participants were instructed to rinse with mouth temperature (35 °C) RO water at least 2 times. Mouth temperature rinse water was used to minimize potential temperature effects, as Green and Frankmann previously demonstrated that bitterness perception varies after cooling the tongue (Green and Frankmann 1987). In total, participants rated the intensity of 12 applications, as each bitterant (quinine and Tetralone) was presented at the 3 loci, once on the right and once on the left.

#### Demographics and measures of beer intake

After completing the spatial taste test, participants answered demographic questions on age, sex, and ethnicity; they also answered questions related to alcohol and beer intake, and type of beer consumed (Table 2). The question responses regarding the frequency of consumption were adapted from NIAAA recommendations. These data were used to explore potential correlations between consumption of pale ales and American-style lagers, and intensity ratings for Tetralone, as iso-alpha-acids are found in much higher concentrations in pale ales than lagers. The questionnaire consisted of the following items: “What types of alcoholic beverages do you/have you ever consumed?” (wine, beer, spirits, other, none), “How often do you consume beer?” (every day, 5–6 days a week, 3−4 days a week, 2 days a week, 1 day a week, 2–3 days a month, 1 day a month, 3–11 days in the past year, 1 or 2 days in the past year, never), “How often do you consume the following types of beer?” (American lagers [e.g., Budweiser, Coors]; stout/porters [e.g., Guinness]; pale ales [IPAs or APAs]; other specialty styles [e.g., Sours, Lambics, etc.]). The initializations IPAs and APAs refer to India Pale Ales, and American Pale Ales, two common craft beer styles in the U.S.; only the initializations were used on the intake questionnaire, as these beers are commonly sold and marketed by their initials in the U.S., and we felt spelling out the full names would only confuse participants. Consumption frequency of each beer type was obtained, using the same frequency options provided for general beer consumption questions. Branching was used, so only participants who had indicated they consume/had ever consumed beer were asked about beer consumption habits. If a participant did not select the beer option under the types of alcohol consumed question, a 0 was recorded for their yearly intake for beer and all beer types.

**Table 2.** Annualized consumption ranges and means (drinking occasions per year) from the self-reported beer questionnaire

Beer type	Annualized mean	Annualized range
American lager	33.5	0–182
Stout/porter	11.7	0–104
Pale ales	19.1	0–182
Other specialty beers	20.7	0–286

### Data analysis

All data from Experiment 2 were analyzed using SAS version 9.4 (Cary, NC). The right and left intensity ratings at each locus for the 2 solutions were averaged for each participant and log transformed prior to analysis (1 point was added to all intensity ratings to resolve the inability to take the log of zero). Differences between test stimuli were tested using repeated measures ANOVA as stated above in Experiment 1. *P*-values were not adjusted for multiple comparisons; however, appropriate Bonferroni corrections are noted in the figure legends. An α level of 0.05 was set a priori for all analyses.

Alcohol and beer intake measures were annualized prior to analysis—that is, a response of 3–4 days per week was recoded as 182/year while 1 day a week was recoded as 52/year. These annualized values were then quarter root transformed to reduce skew, as done previously (Byrnes and Hayes 2013). Regression models via *proc reg* were used to test for relationships between beer intake and log bitterness ratings from the spatial taste test. Sex was not included in the model due to the small sample size after splitting men and women (*n* = 24 each). Visual inspection of separate slopes for men and women (not shown) showed similar patterns, so overall slopes are shown in Figure 5. However, while we did not find any obvious evidence of sex differences, we should also acknowledge we were not powered to look for such effects, as this was an exploratory aim.

**Figure 4.**
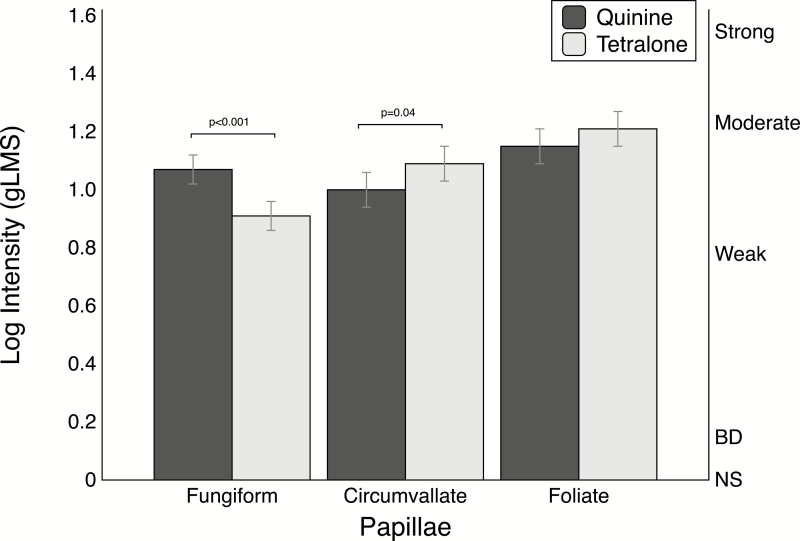
Mean log intensity ratings from the spatial taste test used in Experiment 2. Significance for the *t*-tests between the solutions at each locus is noted. To perform a Bonferroni adjustment for multiple comparisons across the 3 different loci, the *P*-value shown should be multiplied by 3 (e.g., *P* = 0.04 would be adjusted to *P* = 0.12).

**Figure 5.**
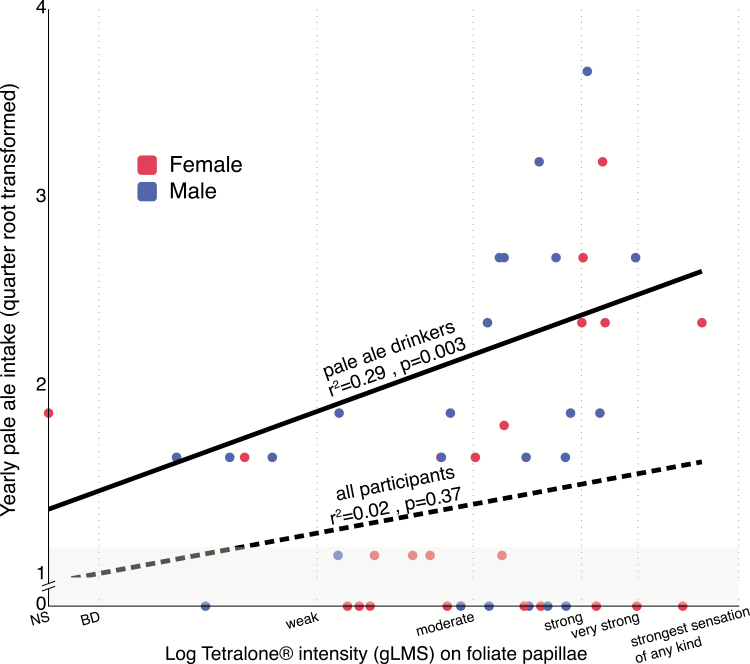
Intensity ratings (logged) for Tetralone on the foliate papillae plotted against self-reported pale ale consumption frequency (annualized, and quarter root transformed) for each participant in Experiment 2. The dashed line represents the correlation across all participants (*n* = 48), while the solid line represents the correlation among regular pale ale consumers (*n* = 28) after removing individuals who reported consumption of pale ales 1–2 times per year or less. These non-consumers (*n* = 20) are shown by the light gray shading in the bottom portion of the graph.

### Experiment 2 results

#### Locus intensity ratings

As in Experiment 1, intensity ratings for the solutions ranged from weak to moderate (Figure 4). Repeated measures ANOVA revealed a significant interaction for locus by stimulus [*F*(2, 94) = 10.38; *P* < 0.001]. Decomposition of the simple effect of stimulus via the *slice* option revealed significant differences between quinine and Tetralone on the circumvallate and fungiform papillae (see Figure 4). Intensity ratings for Tetralone on the front of the tongue (fungiform papillae) were rated lower than the intensity rating for quinine (t=3.79, *P*<0.001). Tetralone was rated higher than quinine on the circumvallate papillae (*t* = 2.11, *P* = 0.04). There was no evidence that the 2 bitterants differed in intensity on the foliate papillae (*t* = 1.41, *P* = 0.16).

The *slice* option also revealed a significant effect of loci for both stimuli (not shown). Tetralone was rated significantly lower on the fungiform papillae compared to the foliate (*t* = 5.16, *P* < 0.001) and circumvallate (*t* = 3.19, *P* = 0.002) papillae. Quinine was rated lower on the circumvallate papillae than the foliate papillae (*t* = 2.48, *P* = 0.01).

#### Self-reported beer consumption and perceived bitter intensity

In the cohort tested here, self-reported beer consumption ranged from 0 beer drinking occasions per year to 286 beer drinking occasions per year with a mean of ~60 beer drinking occasions per year (based on the annualization of the categorical responses described in Demographics and measures of beer intake). Annualized consumption frequency ranges and means for each beer type are shown in Table 2. Three participants reported not consuming alcohol, and 5 additional participants reported not consuming beer.

We originally hypothesized that American-style lager consumption and pale ale (IPA or APA) consumption would show a negative correlation (i.e., beer drinkers would primarily consume either lagers or pale ales, but not both due to the drastic difference in taste profiles [including bitterness], alcohol and calorie content, and factors like price and availability). However, contrary to our expectations, a strong positive correlation between self-reported lager intake and pale ale intake (annualized and quarter root transformed) was observed (*r* = 0.57, *P* < 0.001). That is, in our cohort, we find participants who drink more beer drink more beer, regardless of type.

We also hypothesized that greater intensity ratings for Tetralone would correlate with lower pale ale consumption. The foliate papillae intensity ratings were chosen as the primary measure of intensity for these comparisons as it was the region with the highest mean rating for both quinine and Tetralone. Contrary to our hypothesis, no significant relationship—either positive or negative—was found between perceived Tetralone intensity on the foliate papillae and pale ale consumption when looking across all study participants (*r* = 0.13, *P* = 0.37); this can be seen as the dashed line in Figure 5. However, when looking at only those individuals who regularly consume pale ales (i.e., after excluding the 15 participants who reported zero annual consumption of pale ales and the 5 participants who reported consuming pale ales only 1–2 times per year), a significant relationship was observed, but in the opposite direction of what we had hypothesized. That is, when looking only at regular consumers of pale ales, frequency of pale ale consumption and Tetralone intensity rating on the foliate papillae showed a strong, positive relationship (*n* = 28, *r* = 0.54, *P* = 0.003). To explore whether this significant relationship was simply the result of increased overall bitter perception and increased beer consumption of any beer type, additional regression models were tested (not shown). In regular pale ale consumers (i.e., after excluding participants who reported consumption of no beer or 1–2 beers per year), there was weak evidence of a positive correlation (*n* = 28, *r* = 0.34, *P* = 0.08) between quinine intensity on the foliate papillae and pale ale consumption. Further, no significant relationships were observed between Tetralone intensity on the foliate papillae and overall beer frequency, excluding non-beer drinkers (*n* = 39, *r* = 0.11, *P* = 0.51; an additional participant reported consuming only 1–2 beers per year and was excluded) or reported consumption of American-style lagers, after excluding non-lager drinkers (*n* = 33, *r* = 0.01, *P* = 0.95). Last, we should note that pale ale consumption and Tetralone intensity on the foliate papillae (Figure 5) is shown as a simple linear model. Visual inspection of the plot suggests that a higher order model may be a more appropriate fit, but given the small size, we decided against increasing the number of model parameters to avoid overfitting.

## Discussion

### Loci-based intensity differences

Recent work by Colvin and colleagues investigated differences in intensity rating of sweet, bitter, umami, and “starchy” (maltooligosaccharide) taste stimuli on the fungiform and circumvallate papillae via 2 tasting modes (active and passive) (Colvin et al. 2018). It is important to note that in addition to differences in test stimuli and study design, the present work differs in terms of research question. That is, Colvin and coworkers investigated regional differences within a taste stimulus (i.e., quinine intensity on the front versus back of tongue), whereas our study investigated intensity differences between stimuli at a specific area of the tongue (i.e., quinine intensity was higher than Tetralone on the front of the tongue). In their study, no regional differences were observed for quinine in the passive tasting mode; however, a significant effect was observed between the front and back of the tongue during the active tasting mode. Notably, the null result for the passive taste test is likely due to the lower concentration for quinine. This nuance reinforces the foundational work of Collings (Collings 1974), which suggests regional effects of bitter stimuli are concentration dependent.

Intensity differences between the bitter stimuli on the anterior and posterior tongue may reflect differential responses in receptive fields innervated by CT and GP nerves and/or differential distribution of bitter taste receptors activated by these stimuli. Differential innervation may explain the intensity differences between stimuli of different tastes (i.e., bitter stimuli may elicit a greater response in the GP than sweet stimuli). However, such differences would fail to explain the regional intensity differences between bitter stimuli, as seen here. Previously, Danilova and Hellekant found inconsistent neural response patterns in the CT and GP nerves with different bitter stimuli—that is, quinine had a high GP-to-CT ratio while l-tryptophan had a high CT-to-GP ratio (Danilova and Hellekant 2003). Variable responses in the GP and CT may partially explain the observed differences in bitter intensity from the whole-mouth intensity ratings of Experiment 1 as all locations were simultaneously stimulated. It is possible that the innervation differences between the front and back of the tongue are only critical at perithreshold concentrations, while another factor (perhaps receptor distribution) is more important at suprathreshold concentration levels. Alternatively, this explanation may not apply to all individuals, especially those with taste damage.

In humans, damage to either nerve can increase the oral sensations at other locations in the oral cavity (Bartoshuk et al. 2012). Although we screened for taste damage by excluding those with a self-reported history of ear infections (as well as those with known taste or smell impairments), it remains possible that individuals with varying degrees of unknown taste damage may have participated in the study. To check if taste damage or overall decreased perception on the front of the tongue in some participants might be driving the observed differences in Tetralone and quinine intensity on the front of the tongue in Experiment 2, back to front intensity rating ratios for the quinine and Tetralone ratings were calculated for each participant. The ratios were generated using the logged intensity score of the foliate and fungiform papillae intensity ratings (adding one), and then dividing the foliate by the fungiform ratings. The plot of the quinine ratio versus the Tetralone ratio showed a slight negative trend (not shown), but no significant relationship was observed (*r* = 0.16, *P* = 0.27), indicating that lower overall ratings on the front of the tongue did not drive the differences in intensity seen for quinine and Tetralone or meaningfully influence the results of the study.

Conceivably, differential expression in the bitter taste receptors activated by the stimuli may potentially lead to regional differences in intensity. Tetralone is an humulone isomer, and in heterologous expression models, humulone isomers have been shown to activate TAS2R1, TAS2R14, and TAS2R40 (Meyerhof et al. 2010). Quinine is a more promiscuous stimulus, as in vitro data from similar expression systems indicate it activates 9 different bitter taste receptors: TAS2R4, TAS2R7, TAS2R10, TAS2R14, TAS2R39, TAS2R40, TAS2R43, TAS2R44, and TAS2R46 (Meyerhof et al. 2010). Between the 2 stimuli, only TAS2R14 and TAS2R40 overlap, and the observed intensity difference on the front of the tongue for the 2 stimuli suggests the expression of the receptors may vary within the oral cavity. Thus, reduced or absent expression of TAS2R14 and TAS2R40 on the front of the tongue might be apparent for Tetralone but not quinine, if the additional receptors activated by quinine rescue function on the anterior tongue. However, this explanation depends upon differential expression of the relevant receptors across the tongue, and we are unaware of any data actually showing this in humans.

Still, heterogenous expression of bitter taste receptor genes has been reported previously within papillae. Behrens and others investigated variation of hTAS2R gene expression in human circumvallate papillae using in situ hybridization (Behrens et al. 2007). The number of positive cells was quantified for each hTAS2R and listed as a percentage out of the total number of expressed intragemmal cells to determine differences in expression of the hTAS2R genes. These values ranged from 10.7% to 0.7% of the intragemmal cells. We can infer that the increased expression of certain hTAS2Rs genes on the circumvallate papillae may lead to increased bitter taste intensity on the back of the tongue for bitter compounds that activate the highly expressed receptors. Conversely, these data cannot explain the intensity differences we observed between quinine and Tetralone because the highly expressed hTAS2R genes activated by quinine are equally or more numerous than those activated by Tetralone. The relevance of regional variation in taste receptor gene expression here is restricted to quinine and Tetralone, as SOA still lacks a cognate receptor (Wiener et al. 2012) despite its clearly bitter character.

### Temporal differences between bitter stimuli and loci

The results from the aftertaste intensity ratings of Experiment 1 suggest bitter stimuli differ from one another based on their intensity following expectoration. Here, all bitter stimuli showed a significant decrease in intensity ratings on the anterior tongue, but at the other loci, only quinine showed a significant decrease. Leach and Noble found quinine decayed faster than caffeine during continuous rating after expectoration (Leach and Noble 1986). Here, we did not use caffeine or a continuous rating method; still, the findings of Leach and Noble are roughly consistent with the lower aftertaste ratings we saw for quinine.

The observed temporal effects may be due to differences in the shape and structure of papillae and the affinity of the bitter molecules to bind to the taste receptors on the papillae or with salivary proteins. The structure of the circumvallate and foliate papillae is similar to one another as both papillae contain taste buds in their folds or “trenches” (see Doty 2015 for drawings). The taste buds of the fungiform papillae are located on the top of the papillae structure and do not contain folds. Speculatively, these structural differences may lead to the decreased aftertaste ratings observed on the anterior tongue. The bitter taste on the anterior tongue presumably dissipates faster on fungiform papillae because the bitterant molecules interact with the taste receptors on the exposed surface of the taste bud versus interacting with receptors protected deeper in the folds. The folds in the circumvallate and foliate papillae may retain more bitter molecules after expectoration or swallowing, resulting in lingering bitter perception on the posterior tongue. The structural differences in papillae provide a hypothesis for the loci-based intensity differences; however, this general explanation fails to explain the reduced aftertaste ratings seen for quinine.

Differences in receptor affinity, affinity to salivary proteins, and solubility of quinine in the saliva are additional potential sources of the reduced aftertaste ratings for quinine. Due to differences in hydrophobicity/hydrophilicity, quinine has been shown to react with whey protein at a higher rate than caffeine, resulting in differences in perceived intensity (Tenney et al. 2017). The increased reactivity of quinine with whey protein may also conceivably correspond to increased binding and activity with salivary proteins, leading to reduced amounts available for binding to receptor sites, thereby depressing perceived bitterness. However, this would occur only if quinine’s affinity for salivary proteins were greater than the affinity for the receptor sites. A more detailed examination of the rate of binding between quinine and salivary proteins and the different receptor sites is needed to understand how these interactions may potentially affect the duration of bitter taste.

We should also note that our studies did not use nose clips, and the Tetralone extract used in our study is not odorless. However, at the concentrations we used, no orthonasal odor was noticeable. Additional testing is needed to determine if there is an existing retronasal aroma from Tetralone at the concentrations used here, and whether it influences ratings of bitter intensity via a dumping (Clark and Lawless 1994) or another phenomenon.

### Tetralone intensity ratings and beer intake

Prior research generally suggests that increased bitter perception leads to decreased consumption of bitter food products (Keller et al. 2002; Duffy et al. 2010; Hayes et al. 2015). However, we found some evidence that, at least among pale ale drinkers, those who rated Tetralone as more bitter also reported greater consumption of pale ale style beers. Our results align with recent findings using data from the UK BioBank (Ong et al. 2018). Ong and others found that higher caffeine perception correlated to increased intake of coffee and increased odds of being a heavy coffee drinker. Their finding and the present data suggest that the bitterness from specific compounds in bitter beverages (i.e., iso-alpha-acids in pale ales or caffeine in coffee) may not discourage use, but rather instead reinforce and drive consumption in those who taste the bitterants more intensely. Present work contradicts prior data from Lanier, Hayes, and Duffy on the topic of beer consumption and bitter taste perception (Lanier et al. 2005). However, our study differs from the study of Lanier and colleagues in one key way: it focused on bitterness of 6-n-propylthiouracil (which is not found in the food supply), rather than a hop associated bitterant. It is tempting to speculate that the relatively high alcohol content in pale ales may have previously reinforced liking for hoppy cues via flavor consequence learning. Notably, the study by Ong et al. found that increased propylthiouracil (PROP) sensitivity negatively correlated with alcohol frequency (Ong et al. 2018), which is consistent with Lanier et al. (2005) and other studies (Duffy et al. 2004). Collectively, these reports support the notion that bitterness avoidance is highly dependent on the bitterant used for phenotyping, as well as the bitterants present in a specific food or beverage. Indeed, we found here that bitterness ratings for quinine were also correlated with self-reported pale ale intake, but the variance explained was less than for Tetralone (*r* = 0.34 vs. *r* = 0.54).

It may be possible that some of bitter sensitivity/intake relationships arise from malleable sensitivities developed through recurrent consumption rather than hardwired genetic differences. The effect of repeated exposure on perceived intensity ratings over time appears to be highly dependent on the taste stimuli. Data from mice models show that increased exposure to MSG, saccharin, and NaCl results in the upregulation and expression of the corresponding taste receptors, whereas exposure to quinine stimuli showed an opposite, but not significant effect (Shahbandi et al. 2018). These stimulus dependent outcomes may be due to the caloric/nutritional value and bioactivity of the stimuli. That is, repeated exposure to bitterness would presumably cause upregulation in receptor expression to prevent the ingestion of a potentially toxic dose while sweet receptor expression might be downregulated to increase consumption of energy-dense foods. Research on changes in preferences or perceived intensity after repeated exposure to sweet (Appleton et al. 2018), salty (Bertino et al. 1986), and umami (Noel et al. 2018) stimuli has been reported; however, changes in receptor expression is less studied.

A similar but separate hypothesis suggests that changes in salivary proteins following repeated exposure to bitter stimuli alter bitter taste perception. Research in humans suggests differences in salivary composition between individuals who show hypersensitivity or hyposensitivity to caffeine (Dsamou et al. 2012). Likewise, controlled feeding in rats shows that a diet high in quinine upregulates expression of certain salivary proteins which leads to changes in quinine perception and acceptance (Martin et al. 2018) and detection thresholds (Martin et al. 2019). Alternatively, in humans, repeated exposure to a bitter–sweet beverage resulted in increased pleasantness of the beverage via increased familiarity and associated learning with post-ingestive effects (Stein et al. 2003). Thus, repeated exposure to a bitterant and any subsequent effects on liking/acceptance is presumably the result of multiple factors such as increased familiarity and should not be limited to changes in protein composition. More research is required to determine any causal mechanisms that might underlie the positive relationship observed here between pale ale consumption and the bitterness of Tetralone.

Last, we should also note potential limitations of the beer intake data related to the study environment. Our data were collected on the campus of a large rural land-grant university, and a subset of our participants were undergraduates. Beer consumption in undergraduate students is presumably influenced strongly by factors beyond perception of hop extracts; while this might explain why we failed to find an inverse relationship between pale ale and lager consumption, it cannot explain the positive relationship between bitterness and pale ale consumption frequency. Also, we limited our intake questions to beer. That is, intake of tonic water was not assessed because we did not believe it would be widely consumed among our sample population. This limits our ability to generalize beyond beer; future studies should include more bitter foods and beverages.

### Comparison of data collection methods/study protocol

Both approaches used here (whole-mouth and spatial taste test) provided similar results irrespective of the delivery method or the cognitively different tasks required of participants when rating the stimuli. The sip and spit protocol in Experiment 1 asked participants to focus on a specific locus in the mouth while rating intensity. To do this, participants had to separate the perception at the locus being rated from intensities at other loci that were stimulated simultaneously. Shikata and others previously demonstrated that taste lateralization occurs on the front of the tongue without interference of discriminative tactile cues, as participants were able to laterally discriminate a tastant from a blank stimulus (Shikata et al. 2000). Their findings appear to support our contention that participants would be able to localize the perceived intensity of the test stimuli in Experiment 1. However, caution is still warranted, as substantial differences between the 2 stimulus delivery systems prevent strong conclusions in this regard.

One limitation of the protocol for Experiment 1 comes from the reported intensity on the lip and middle of the tongue, which do not contain taste receptors. Although the mean lip intensity ratings were all below weak, a significant difference between the in-mouth and aftertaste ratings on the lip was observed for quinine. This occurrence and the high ratings on the middle of the tongue is likely due to the mislocalization of taste where the tactile sensations can lead to the mislocalization of taste (see Todrank and Bartoshuk 1991; Delwiche et al. 2000; Green 2003; Lim and Green 2008). This phenomenon is more likely to occur in a sip and spit protocol as the swishing and spitting portion of the taste procedure increases the tactile sensations in the oral cavity leading to possible mislocalization. Notably, Lim and Green reported that that mislocalization was more likely for bitter stimulus than sweet stimulus (Lim and Green 2007, 2008). Separately, the similar intensity ratings on the middle and back of tongue may simply be the result of confusion between the 2 areas. That is, the location of the middle of the tongue may not have been clear to participants, and participants likely used the same intensity ratings for the back and middle of the tongue. This suggests that future research using a similar protocol should either provide clear instructions for the rated areas, or use a more limited ballot that omits intensity ratings on the middle of the tongue.

In contrast to the sip and spit procedure, the spatial taste test in Experiment 2 was a simpler task, requiring less attention and fewer cognitive demands as only one region was stimulated at a time. Experiment 2 also provided more control as the experimenter applied the stimuli to individual tongue regions. This revealed differences between Tetralone and quinine on the back of the tongue between the circumvallate and foliate papillae that were not possible in the sip and spit study. Still, Experiment 1 was much more time efficient than Experiment 2 and allowed for the collection of a larger sample size with substantially decreased experimenter time. Given the benefits and shortfalls of each approach, we believe the 2 approaches complement each other, and contribute to improved understanding of regional variation in taste perception. The sip and spit paradigm is appropriate for exploratory data collection to identify differences between stimuli, and the spatial taste test is well suited for follow-up studies to confirm observed differences in a more controlled manner.

## Conclusions

Bitter stimuli vary in perceived intensity at various locations in the oral cavity, as intensity differences were seen in both a spatial taste test and a sip and spit protocol. Bitter stimuli also differ in terms of temporal perception, as some stimuli tended to linger while others diminished much more quickly after reaching the same maximum intensity. Additional research is needed to determine the cause of the regional variation in perceived intensity and the variation in aftertaste intensity and duration between bitter stimuli. Further, the intensity of bitter stimuli found in pale ale beer (i.e., the iso-alpha acid hop extract Tetralone) was positively correlated to self-reported consumption frequency of pale ales. Additional research is needed to determine the causal direction, if any, for this relationship.

## Supplementary material

Supplementary data are available at *Chemical Senses* online.

Supplementary Figure 1 Mean log intensity ratings and standard error of the mean for in-mouth ratings at different oral loci to represent the locus effect from Experiment 1. Means that share a letter within a stimulus do not significantly differ.

Supplementary Figure 2 Same as Supplementary Figure 1, but for aftertaste intensity ratings made after spitting out the solution and waiting 15 s. Means that share a letter within a stimulus do not significantly differ.

Supplementary Figure 3 Diagram of the tongue showing the various loci stimulated in Experiment 2.

Supplementary Figure 4 Example photographs showing how the tongue was manipulated by the participant in Experiment 2 to allow the experimenter access to the various tongue regions.

bjz064_suppl_Supplementary_Figure_1

bjz064_suppl_Supplementary_Figure_2

bjz064_suppl_Supplementary_Figure_3

bjz064_suppl_Supplementary_Figure_4

bjz064_suppl_Supplementary_Material
