# Supplementary figures and images for: Regional Variation of Bitter Taste and Aftertaste in Humans
Source: Chem Senses. 2019 Sep 21;44(9):721–32. doi: 10.1093/chemse/bjz064 (PMC6872973; doi:10.1093/chemse/bjz064)

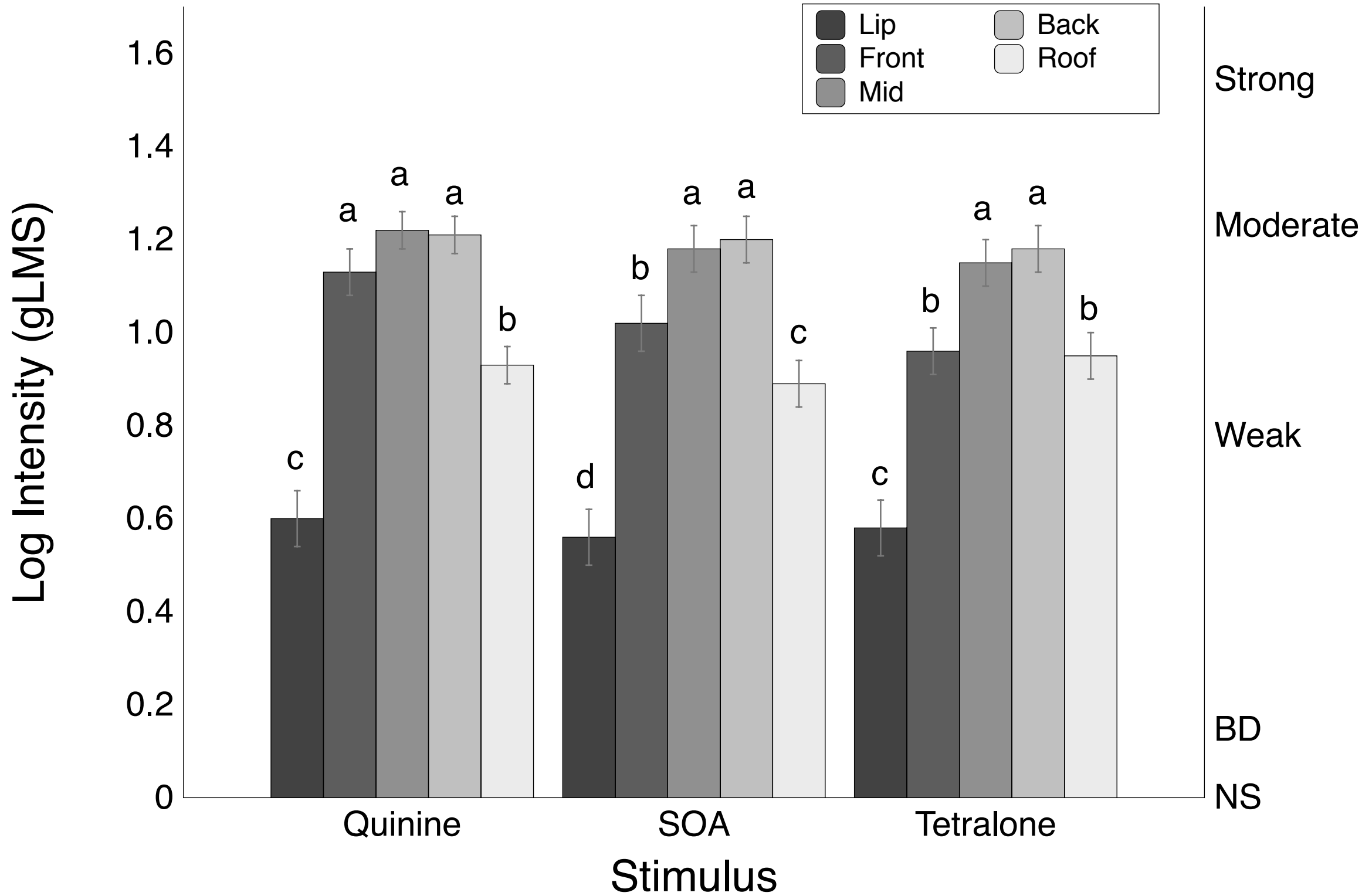

Supplement: bjz064_suppl_Supplementary_Figure_1 [file bjz064_suppl_supplementary_figure_1.pdf]

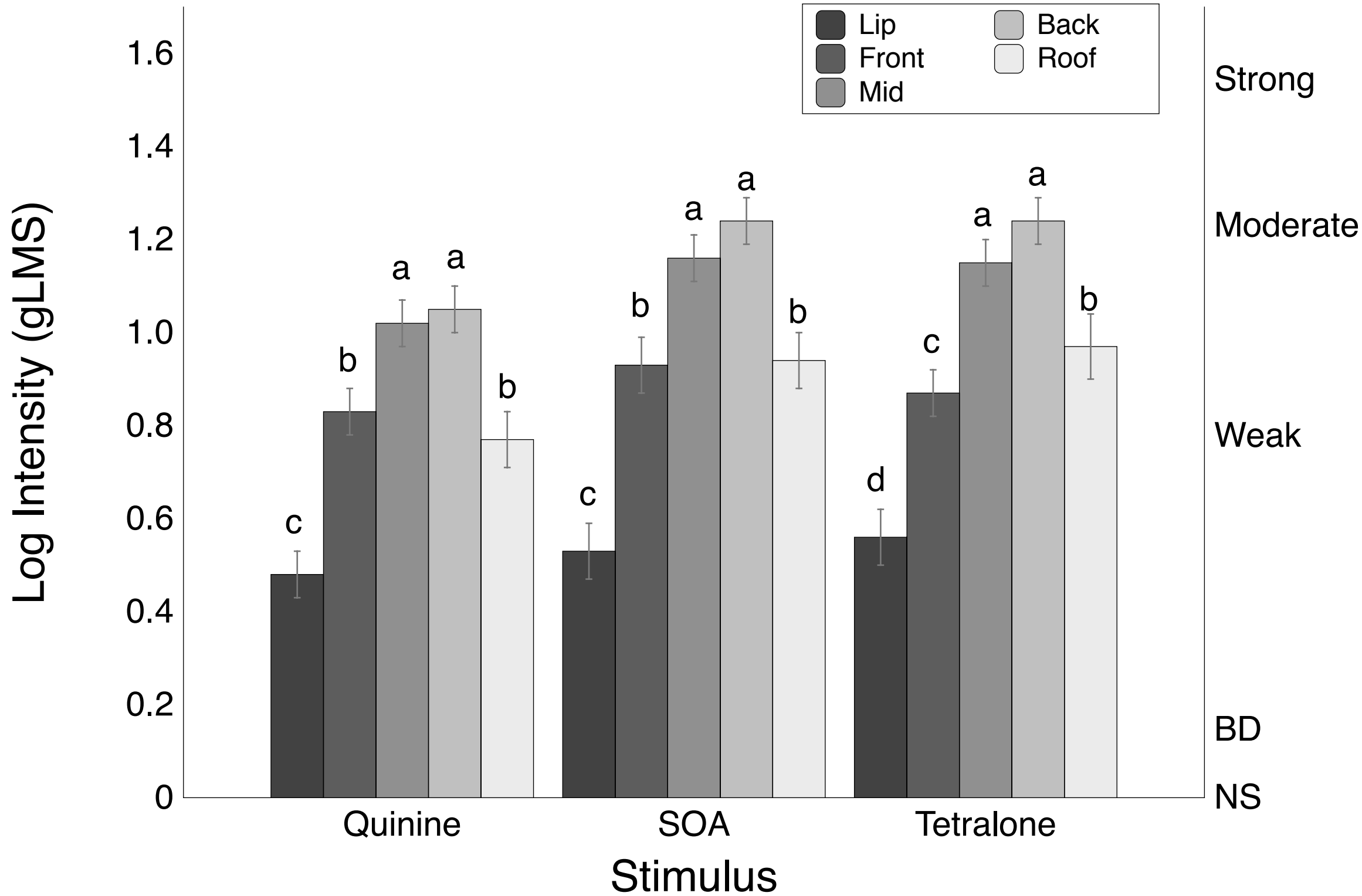

Supplement: bjz064_suppl_Supplementary_Figure_2 [file bjz064_suppl_supplementary_figure_2.pdf]

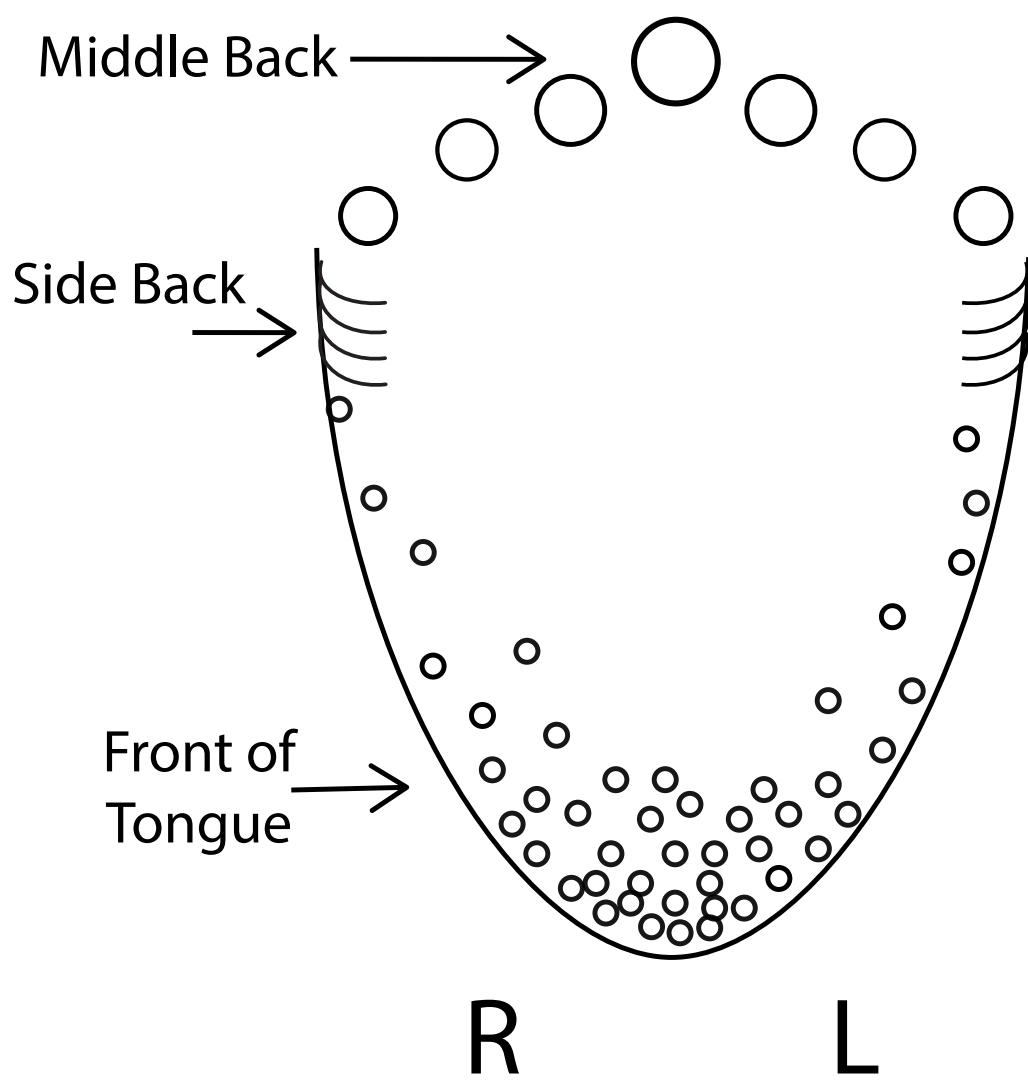

Supplement: bjz064_suppl_Supplementary_Figure_3 [file bjz064_suppl_supplementary_figure_3.pdf]
