# Supplementary material for: Regional Variation of Bitter Taste and Aftertaste in Humans
Source: Chem Senses. 2019 Sep 21;44(9):721–32. doi: 10.1093/chemse/bjz064 (PMC6872973; doi:10.1093/chemse/bjz064)
Supplement: bjz064_suppl_Supplementary_Figure_4 [file bjz064_suppl_supplementary_figure_4.pdf]

Front Rating:

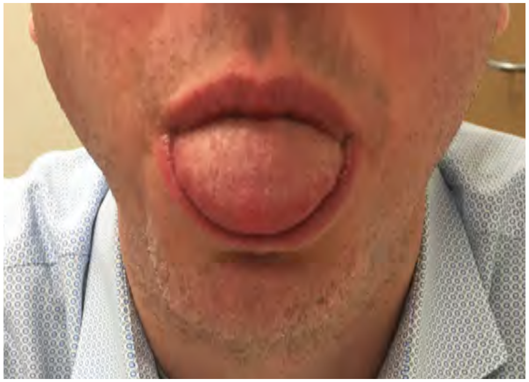

Middle Back Rating:

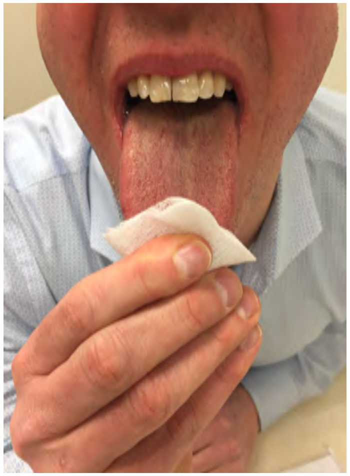

Side Back Rating:

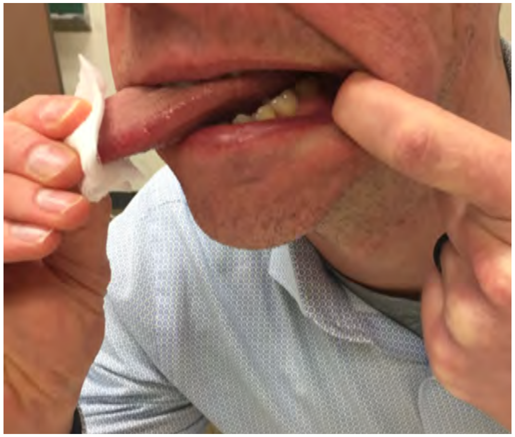

Images shown here are of the corresponding author (not a participant) and are provided with his permission.
