# Supplementary material for: Regional Variation of Bitter Taste and Aftertaste in Humans
Source: Chem Senses. 2019 Sep 21;44(9):721–32. doi: 10.1093/chemse/bjz064 (PMC6872973; doi:10.1093/chemse/bjz064)
Supplement: bjz064_suppl_Supplementary_Material [file bjz064_suppl_supplementary_material.docx]

**Supplemental Figure 1:** Mean log intensity ratings and standard error of the mean for in-mouth ratings at different oral loci to represent the locus effect from Experiment 1. Means that share a letter within a stimulus do not significantly differ.

**Supplemental Figure 2:** Same as Supplementary Figure 1, but for aftertaste intensity ratings made after spitting out the solution and waiting 15 seconds. Means that share a letter within a stimulus do not significantly differ.

**Supplemental Figure 3:** Diagram of the tongue showing the various loci stimulated in Experiment 2.

**Supplemental Figure 4:** Example photographs showing how the tongue was manipulated by the participant in Experiment 2 to allow the experimenter access to the various tongue regions.
